# LMP1^+^SLAMF1^high^ cells are associated with drug resistance in Epstein-Barr virus-positive Farage cells

**DOI:** 10.18632/oncotarget.15600

**Published:** 2017-02-21

**Authors:** Heejei Yoon, Young Hyeh Ko

**Affiliations:** ^1^ Clinical Research Center, Sungkyunkwan University School of Medicine, Seoul, Korea; ^2^ Department of Pathology, Samsung Medical Center, Sungkyunkwan University School of Medicine, Seoul, Korea

**Keywords:** EBV+ DLBCL, LMP1, SLAMF1, CHOP, NF-κB

## Abstract

How Epstein-Barr virus (EBV) affects the clinical outcome of EBV-positive diffuse large B-cell lymphoma (DLBCL) remains largely unknown. The viral oncogene LMP1 is at the crux of tumorigenesis and cell survival. Therefore, we examined the association between LMP1^high^ cells drug resistance. We first assessed SLAMF1 as a surrogate marker for LMP1^high^ cells. LMP1 and its target gene CCL22 were highly expressed in SLAMF1^high^ Farage cells. These cells survived longer following treatment with a combination of cyclophosphamide, doxorubicin, vincristine and prednisone (CHOP). Genes associated with interferon-alpha, allograft rejection, NF-κB and STAT3 were also overexpressed in the surviving Farage cells. Specifically, CHOP treatment increased IL10, LMP1 and pSTAT3 expression levels in a dose-dependent fashion. Addition of exogenous IL4 greatly increased the levels of LMP1 and pSTAT3, which rendered the Farage cells more resistant to CHOP by up-regulating the anti-apoptotic genes BCL-XL and MCL1. The Farage cells were sensitive to Velcade and STAT3, 5, and 6 inhibitors. Inhibition of NF-κB and STAT3, in combination with CHOP, decreased LMP1 levels and effectively induced cell death in the Farage cells. We suggest that LMP1^high^ cells are responsible for the poor drug response of EBV+ DLBCL and that perturbation of the NF-κB and STAT signaling pathways increases toxicity in these cells.

## INTRODUCTION

Epstein-Barr virus (EBV)-positive (+) diffuse large B-cell lymphoma (DLBCL) accounts for 4–11% of all cases of DLBCL [1–3]. EBV+ DLBCL exhibits relatively few genetic alterations compared with EBV- DLBCL [4]. However, EBV+ DLBCL is associated with poor prognosis and a median survival of 2 years in Asian populations [1–2, 5–8]. Viral oncogenes and immunosuppression have been suggested to play important roles in tumorigenesis [9]. However, precisely how the presence of EBV affects current chemotherapies in EBV+ DLBCL remains largely unknown.

Most cases of EBV+ DLBCL, particularly in Asia, have shown an activated B cell (ABC)-like immunophenotype, which is consistent with the substantial NF-κB activation that was revealed by immunohistochemistry and gene expression profiles [4, 8, 10–11]. NF-κB activation is a common feature that occurs through various mechanisms in ABC-DLBCL. Somatic mutations in *TRAF3*, *CARD11*, *CD79A/B*, *TNFAIP3/A20* and *MYD88*, which activate the NF-κB signaling pathway, were identified in EBV- DLBCL [12–15]. Although a deletion mutation in *TNFAIP3* has been reported in 1 of 8 EBV+ DLBCL samples tested [16], other mutations associated with NF-κB activation have yet to be identified in EBV+ DLBCL. Among the EBV latent genes, LMP1 expression may account for the oncogenic activation of NF-κB in EBV+ DLBCL. LMP1 is a transmembrane protein that constitutively activates both classical and alternative NF-κB signaling pathways without ligands [17–18]. LMP1 also activates the p38, JNK, mitogen-activated protein kinase (MAPK), phosphatidylinositol 3-kinase (PI3K), IRF7, and STAT pathways [18–19]. In a mouse model, transgenic expression of LMP1 in B cells was sufficient to develop lymphoma that resembled human DLBCL under immunosuppression, which mimics EBV+ DLBCL [9]. Since LMP1 has been demonstrated to increase drug resistance in lymphoma cells [20], LMP1-driven constitutive NF-κB activation, in cooperation with the multifunctional effect of LMP1 on diverse signaling pathways, may be responsible for the poorer prognosis of EBV+ DLBCL.

LMP1 expression can be induced *in vitro*, in the absence of the viral trans-activators EBNA2, by cytokines such as IL10, IL13 and IL4 [21–22]. LMP1 induction by these cytokines is mediated by the STAT3 or STAT6 signaling pathways. Reversely LMP1 also induces IL10 in EBV-infected B cells, which forms an autocrine feed-forward regulatory loop between IL10 and LMP1 [23–24]. IL10 is involved in the proliferation and autonomous growth of EBV-transformed B cells [25–26]. Histologically, EBV+ tumor cells in EBV+ DLBCL have been detected within a background of extensive infiltrating immune cells, including histocytes, mast cells, and T cell subsets [27]. Previously, we identified the overexpression of several T cell-recruiting chemokines in EBV+ DLBCL [4]. Oyama et al. revealed the co-expression of LMP1 and the chemokines CCL17 and CCL22 and found that LMP1+ tumor cells were closely surrounded by CCR4^+^ Th2 cells and regulatory T cells [28]. This finding implies that the cytokines IL4 and IL10 that are secreted by the recruited T cells can modulate the expression levels of LMP1 in tumor cells. Therefore, the tumor microenvironment may support the drug resistance and immune evasion of LMP1+ cells in EBV+ DLBCL.

Currently, patients diagnosed with EBV+ DLBCL are treated with an anthracycline-based combination chemotherapy consisting of cyclophosphamide, doxorubicin, vincristine, and prednisone (CHOP). The addition of the CD20 monoclonal antibody rituximab to CHOP treatment has been tested, however no significant improvements have been observed [5, 7]. How the EBV+ DLBCL cells respond to CHOP therapies and the underlying mechanism of CHOP resistance remains largely unknown. In this study, we isolated cells with high levels of LMP1 expression by using the cell surface marker SLAMF1 (CD150) and analyzed the gene expression profiles of CHOP-resistant cells. We also tested the effect of IL4 on CHOP resistance. Finally, we assessed the synergistic effect of inhibiting phosphorylated STATs in combination with an NF- κB inhibitor.

## RESULTS

### LMP1 and CCL22 are highly expressed in SLAM ^high^ Farage cells

Farage cells are an EBV+ DLBCL cell line that was derived from a 70-year-old woman [29]. Farage cells are considered a CD20- and CD19-positive germinal center B cell-like (GCB) type of lymphoma [30]. To confirm the cell of origin for the Farage cells, we analyzed the expression of BCL6, IRF4/MUM1, and CD10 by using fluorescence-activated cell sorting (FACS). We included the three cell lines Raji, Toledo and IM-9 as controls. More than 50% of Farage cells were BCL6 and IRF4 positive and CD10 negative in Farage cells (Supplementary Figure 1). Based on Han's algorithm [31], this finding suggests that most of the Farage cells are a non-GCB type.

**Figure 1:**
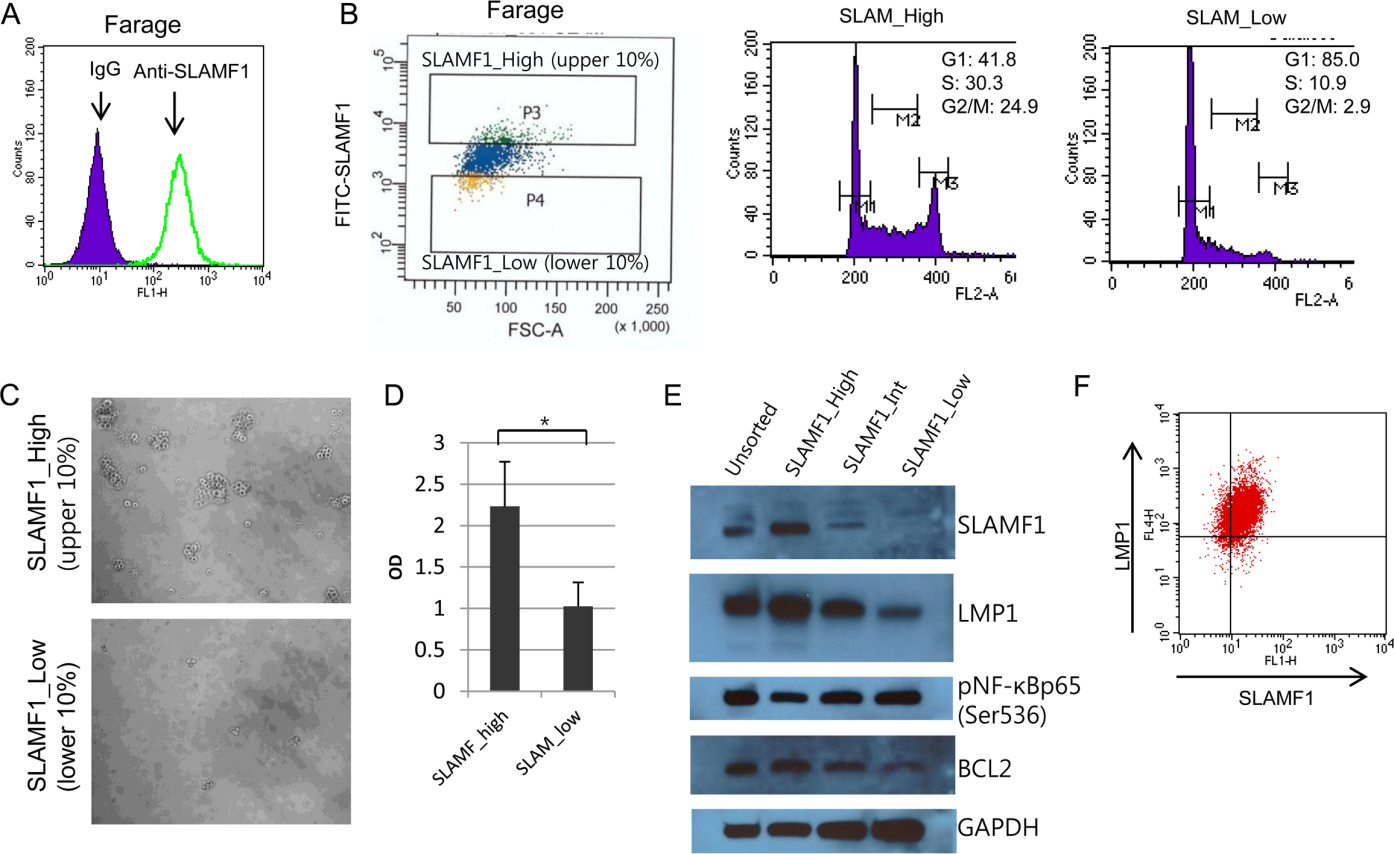
LMP1 is concordantly expressed with SLAMF1 in Farage cells (**A**) SLAMF1 expression at the cell surface of Farage cells. (**B**) Farage cells were labeled with FITC-anti-SLAMF1 and the top and bottom 10% of the labeled cells were sorted. The cell cycle distribution in each group was analyzed. (**C**) The sorted SLAMF1^high^ and SLAMF1^low^ cells were seeded and cultured for 2 weeks. Larger cell clumps were observed in the SLAMF1^high^ cells compared with the SLAMF1^low^ cells, which suggested that the SLAMF1^high^ cells have more regrowth potential. (**D**) Cell proliferation was also assessed with a cell viability test using the WST-1 reagent. The O.D. score correlates with viable cell numbers. The higher O.D. score for the SLAMF1^high^ cells supported our hypothesis that the cell clumps resulted from cell proliferation rather than simple cell aggregation (*p < 0.05). (**E**) Western blotting for SLAMF1, LMP1, phosphor-NF-κB p65 (Ser536), and BCL2. GAPDH served as a loading control.

The expression level of LMP1 varies widely between individual EBV-infected tumor cells *in vivo* and *in vitro* [28, 32]. We hypothesized that at the level of the single cell, EBV+ cells exhibit differenttial drug responses that are correlated with LMP1 expression. To isolate live cells with differential LMP1 levels, a cell surface marker associated with LMP1 expression levels was required because there are no available commercial anti-LMP1 antibodies to isolate LMP1-positive live cells. SLAMF1 was highly induced in EBV infected lymphoblastoid cells [33] and induced by ectopic expression of LMP1 through the NF-κB signaling pathway [34]. We therefore tested whether SLAMF1 expression levels correlated with LMP1 expression levels. SLAMF1 is expressed on the cell surface of almost all Farage cells (Figure 1A). Then, we sorted the top, middle and bottom 10% of the Farage cells according to SLAMF1 expression levels (Figure 1B). We labeled these fractions SLAMF1^high^, SLAMF1^inter^ and SLAMF1^low^. The average cell size of the SLAMF1^high^ cells was larger than that of the SLAMF1^low^ cells. Approximately 55% of the SLAMF1^high^ cells were in the S/G2/M phase of the cell cycle, whereas most SLAMF1^low^ cells were in the G1 phase (Figure 1B). When cultured in growth media, SLAMF1^high^ cells repopulated faster than SLAMF1^low^ cells (Figure 1C and 1D). This result indicates that high SLAMF1 expression is associated with continued cell proliferation and/or cell cycle progression. We next performed western blotting for SLAMF1, LMP1, phosphorylated NF-κB p65 (Ser536), and BCL2 (Figure 1E). SLAMF1 was most abundant protein in the SLAMF1^high^ cells, which was concordant with the LMP1 and BCL2 expression levels, but phosphorylated NF-κB p65 did not correlate with the LMP1 levels. To test whether cell surface expression of SLAMF1 correlates with LMP1 expression at the single cell level, we fixed and sorted cells after labeling them with primary and secondary antibodies. As shown in Figure 1F, SLAMF1 expression levels are correlated with those of LMP1.

**Figure 2:**
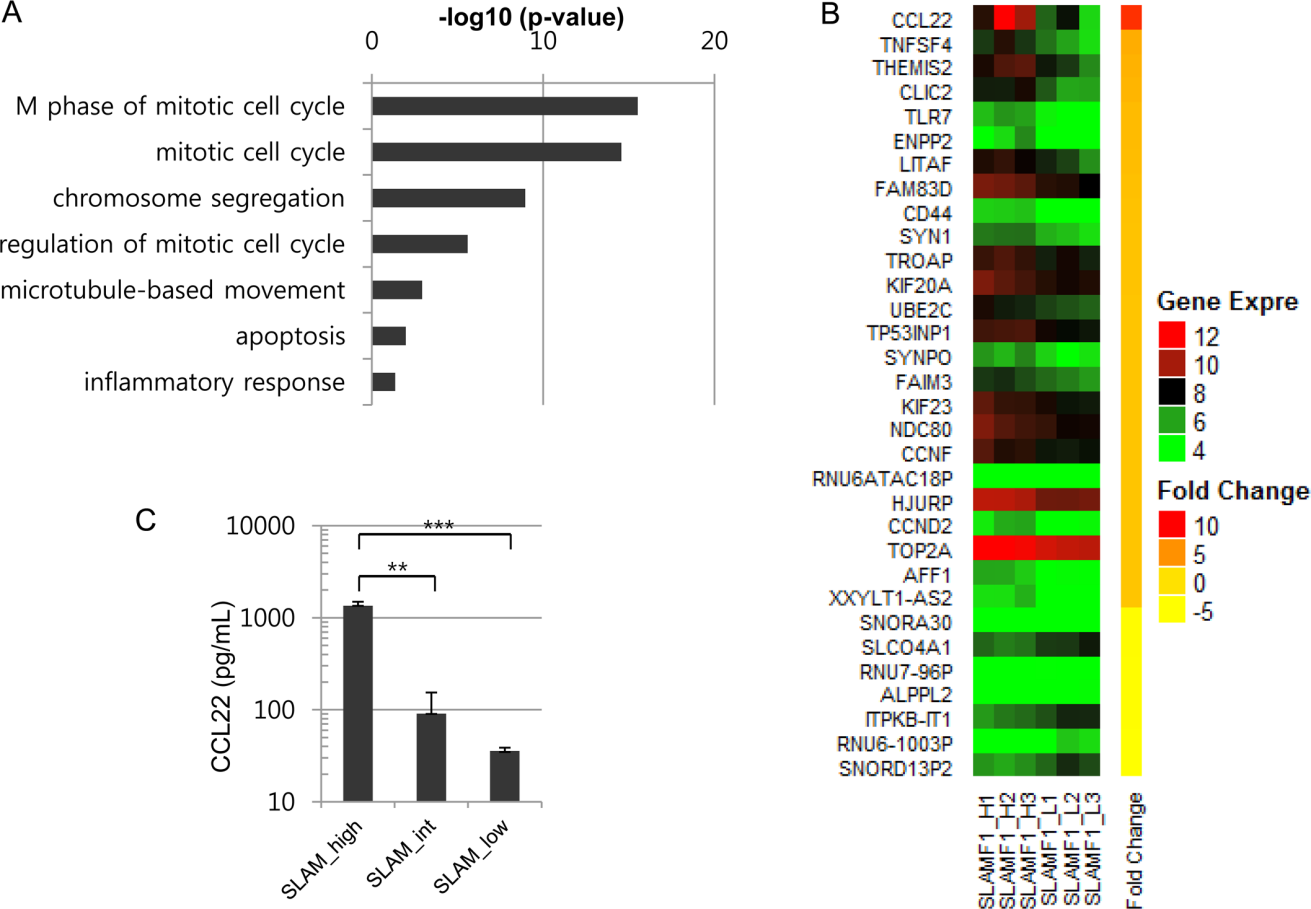
CCL22 is overexpressed in SLAMF1^high^ Farage cells (**A**) Gene set analysis was performed with differentially expressed genes (DEGs) between the SLAMF1^high^ and SLAMF1^low^ Farage cells. Genes associated with the mitotic cell cycle were enriched among the overexpressed genes in the SLAMF1^high^ Farage cells. (**B**) The top DEGs. CCL22 was overexpressed by 9-fold in the SLAMF1^high^ Farage cells. (**C**) CCL22 expression was confirmed by ELISA (**p < 0.001, ***p < 0.0001).

We next compared the gene expression profiles of SLAMF1^high^ cells and SLAMF1^low^ cells. The GO term analysis of the differentially expressed genes suggested that most of the enriched gene sets are associated with the mitotic cell cycle and chromosome segregation Figure (2A and Supplementary Table 1), which were consistent with the results observed in the cell cycle analysis (Figure 1B). Genes associated with inflammation were enriched in the SLAMF1^high^ cells, including CCL22, TNFSF4, IL2RA, CD44 and TLR7 (Supplementary Table 1). Especially, CCL22, a chemokine that attracts T cells expressing CCR4 including regulatory T cells (Tregs), showed the highest fold changes (Figure 2B). We confirmed the elevated expression of CCL22 in SLAMF1^high^ cells by ELISA (Figure 2C). Our results are consistent with a previous report [28] showing that CCL22 is co-expressed in diffuse large B cells that express high levels of LMP1 *in vivo*. Collectively, the expression level of SLAMF1 at the cell surface functionally correlates with the LMP1 level in a single cell, and the LMP1^high^ cells are enriched in the SLAMF1^high^ Farage cell fraction.

### SLAMF1^high^ cells are more resistant to CHOP treatment

**Figure 3:**
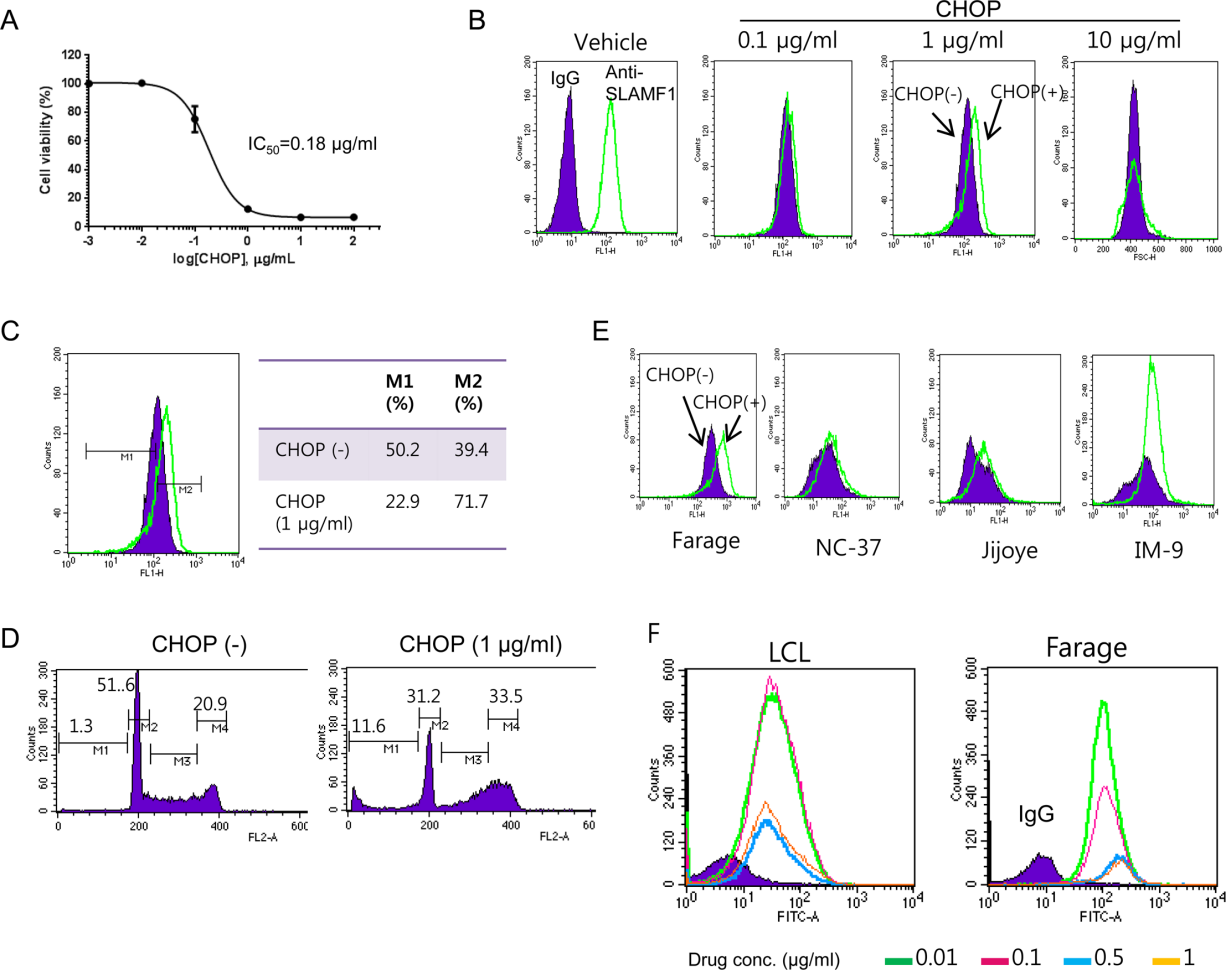
SLAMF1^high^ Farage cells survived CHOP treatment (**A**) Determination of IC_50_ of CHOP in the Farage cells. (**B**) An increase in the SLAMF1^high^ Farage cells occurred at 2 days after CHOP treatment, which was apparent at a concentration of 1 μg/mL CHOP (**C**). (**D**) The SLAMF1^high^ cells were enriched while cell death was ongoing. The farage cells were exposed to 1 μg/mL CHOP for 2 days followed by FACS analysis. (**E**) The SLAMF1^high^ cells in other cell cancer cell lines were increased after CHOP treatment but not in the lymphoblastoid cell lines (LCL) (**F**).

Ectopic expression of LMP1 renders cells more resistant to anti-cancer drugs [20]. LMP1 was highly expressed in SLAMF1^high^ Farage cells (Figure 1E); therefore, we assessed whether the SLAMF1^high^ cells are enriched after CHOP treatment. We treated Farage cells with various doses of CHOP for 3 days and determined their cell viability with the WST-1 reagent (Figure 3A). The IC_50_ of CHOP in the Farage cells was approximately 0.18 μg/ml. We then treated the Farage cells with three doses of CHOP for 2 days and analyzed the expression level of cell surface SLAMF1 by FACS (Figure 3B). SLAMF1 was clearly shifted toward a higher expression level (up to 1 μg/ml CHOP), but most cells died at concentration of 10 μg/ml CHOP (Figure 3B and 3C). CHOP interfered with the G1/S transition, and cells at G2/M phase accumulated (Figure 3D). A subG1 fraction of cells representative of apoptotic cells were increased (Figure 3D). Likewise, SLAMF1^high^ cells seemed to be enriched after CHOP treatment in EBV+ Jijoye and IM-9 cells (Figure 3E). However, the same pattern was not observed in a lymphoblastoid cell line (LCL) (Figure 3F), which suggested that the enrichment of SLAMF1^high^ cells by CHOP might be tumor specific.

**Figure 4:**
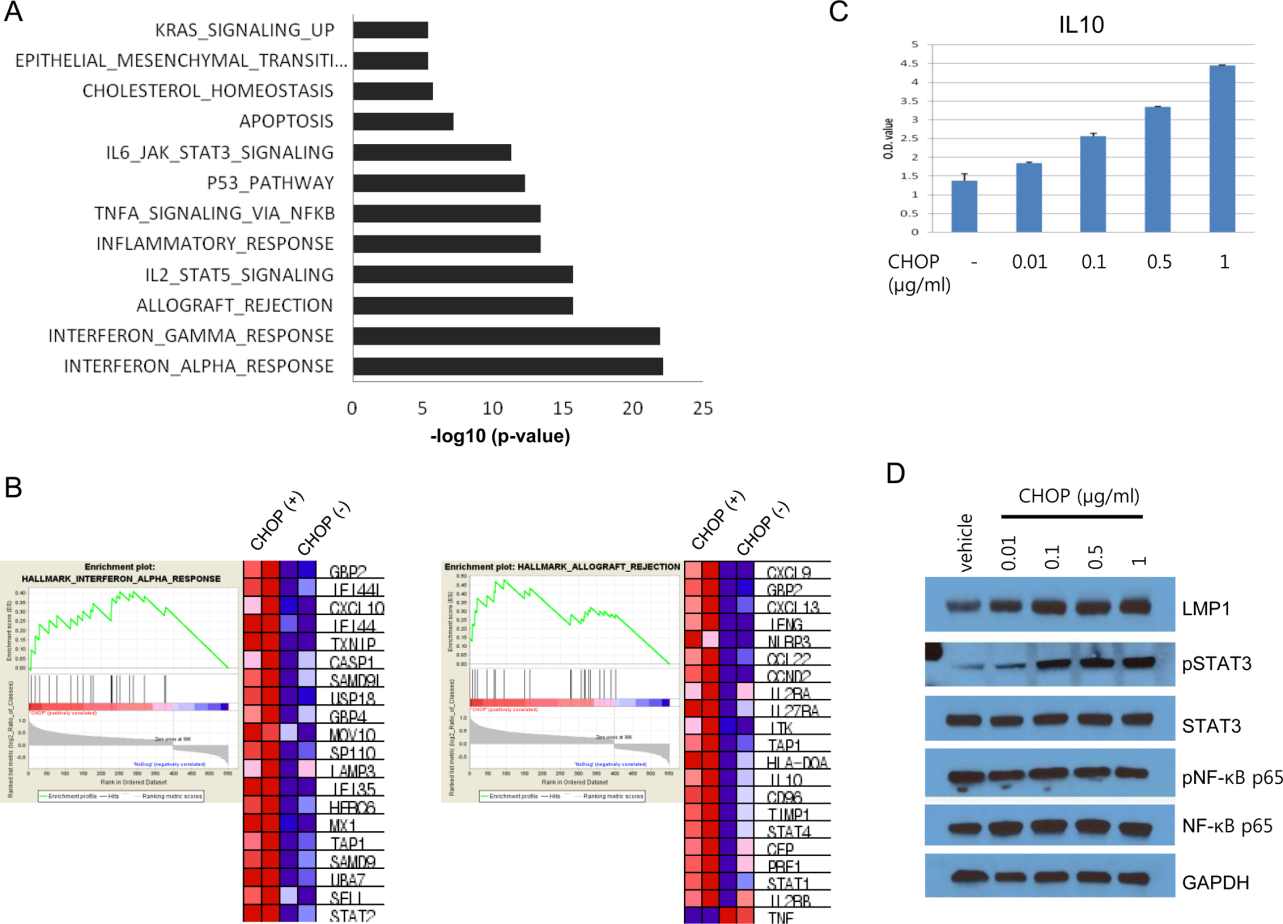
Gene set analysis of CHOP-surviving cells and IL10 expression (**A**) Differentially expressed genes between the CHOP-treated and untreated Farage cells were applied to the Molecular Signatures Database (MSigDB) to identify significantly enriched gene sets. Genes associated with the interferon-alpha response or interferon-gamma response were the most significantly enriched among the DEGs. (**B**) List of genes associated with the interferon-alpha response and allograft rejection. The expression levels were represented by the heatmap, where the red and blue squares indicate over- and underexpression, respectively. (**C**) The Farage cells were seeded in a T25 flask, and the next day, the cells were treated with the indicated concentrations of CHOP for 24 h. CHOP-treated cells were washed and seeded again in a 96-well plate for 24 h without CHOP. The culture media were removed, and ELISA was performed using an IL10 ELISA kit. (**D**) The Farage cells were treated with the indicated doses of CHOP followed by SDS-PAGE and western blotting.

To examine the molecular features of the cells that survived CHOP treatment, we generated gene expression profiles using microarrays. We analyzed the significantly enriched gene sets and pathways in CHOP-treated Farage cells compared with non-treated Farage cells. Overexpressed gene sets in the CHOP-treated cells are involved in interferon-alpha or interferon-gamma responses and allograft rejection (Figure 4A and Supplementary Table 2). Especially, STAT2, TAP1, CXCL10, GBP4, CASP1, TXNIP, IFI44L, MX1, IFI44, USP18, and SAMD9L were commonly overexpressed among the genes associated with both interferon-alpha and interferon-gamma (Supplementary Table 2). Genes associated with NF-κB and the STAT3 and 5 signaling pathways were also overexpressed (Figure 4A and Supplementary Table 2). Specifically, of the genes regulated by NF-κB in response to TNF-α, CXCL10, CD69, IL7R, EDN1, NFIL3, TAP1, TNFAIP2, JUN, PLK2, DRAM1, CFLAR, AREG, PLAU, DUSP5, DUSP1, KLF2, SIK1 and B4GALT5 were overexpressed (Supplementary Table 2). Not only were STATs 1, 2 and 4 overexpressed, but genes associated with the STAT3 and 5 signaling pathways were also overexprssed, which suggested that these STAT signaling pathways were activated (Figure 4A and Supplementary Table 2). Interestingly, IRF4, PRDM1 and PRKCB expression levels increased, and BCL6, MYC, EGR1 and NR4A3 expression decreased in CHOP-treated cells. These genes are part of the discriminators that distinguished the fatal or refractory DLBCL from the cured DLBCL [35]. Of note, IL10 mRNA increased more than 2-fold following CHOP treatment. Because IL10 forms an autocrine feed-forward loop with LMP1, we tested whether the CHOP treatment increases IL10 secretion and LMP1 levels. We treated Farage cells with CHOP and performed an ELISA and western blot. IL10 secretion was indeed increased in a dose-dependent manner, and LMP1 levels were also proportionally increased (Figure 4C and 4D). The levels of pSTAT3 were also increased after CHOP treatment, in agreement with our gene set analysis described above (Figure 4D).

### IL4 protects Farage cells from CHOP-induced cell death

**Figure 5:**
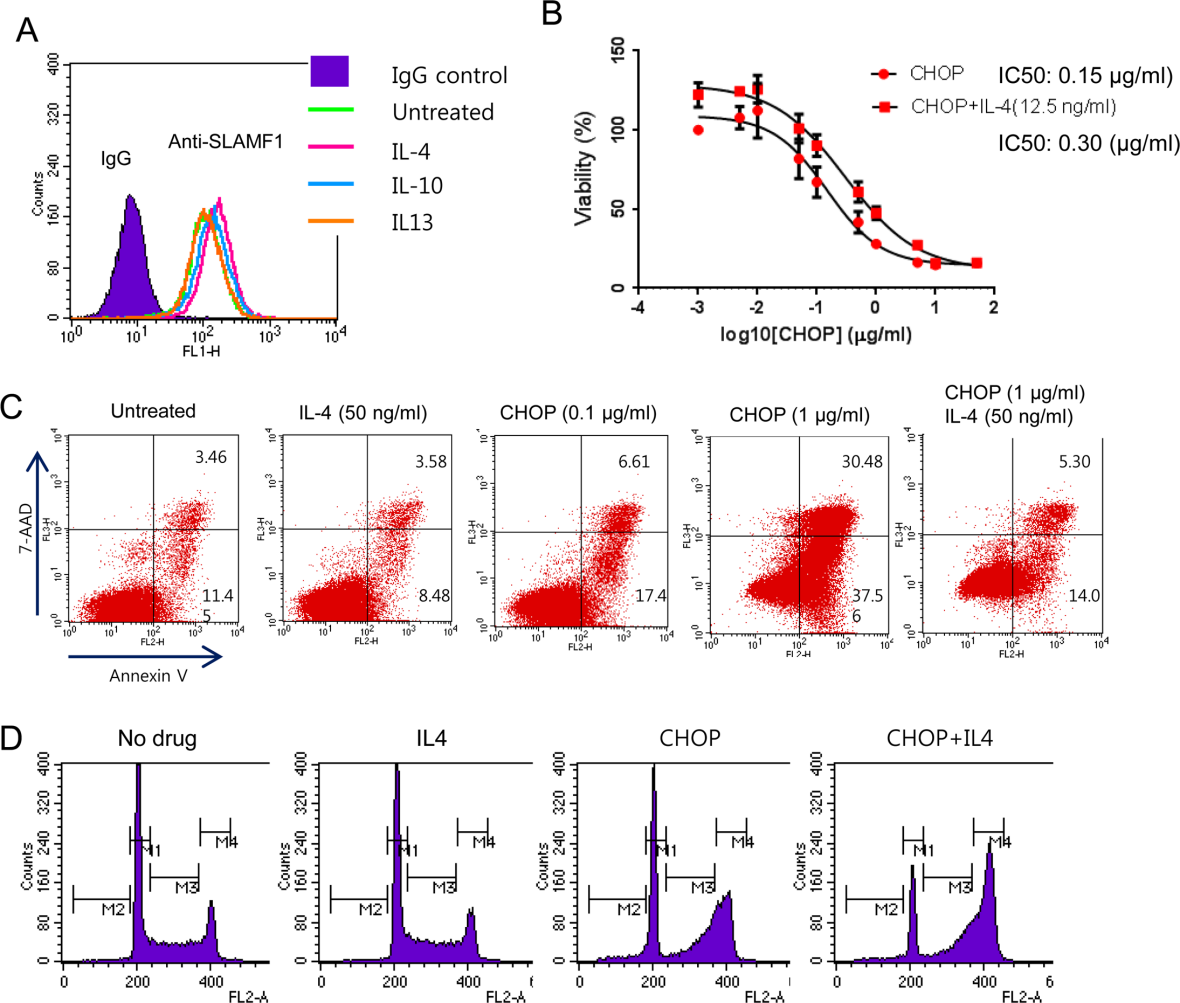
Exogenous IL4 conferred increased resistance to CHOP in Farage cells (**A**) The cytokines IL4 (50 ng/mL) or IL10 (50 ng/mL) increased SLAMF1 levels in the Farage cells. (**B**) The addition of IL4 made the Farage cells more resistant to CHOP. (**C**) IL4 inhibited apoptosis triggered by CHOP. The Farage cells were treated with 0.1 or 1 μg/mL CHOP alone or in combination with IL4 (50 ng/mL). (**D**) The addition of IL4 to CHOP arrested the Farage cells in the G2/M phase.

IL4 can enhance B cell survival and proliferation [36]. In our previous gene expression study we observed overexpression of downstream target genes of IL4 in EBV+ DLBCL [4]. CCL22, which is overexpressed in SLAMF1^high^ and CHOP-treated cells, recruits IL4-secreting Th2 cells [28]. Furthermore, IL4 stimulates LMP1 expression through STAT6 [21]. Therefore, it is possible that in a tumor microenvironment IL4 will render cancer cells more resistant to CHOP treatment. To address this hypothesis, we first examined whether the addition of IL4 to culture medium affects the expression levels of SLAMF1 at the cell surface by FACS analysis. We treated Farage cells with IL4, IL13 and IL10 for 24 h before FACS analysis. IL4 and IL10 treatment increased SLAMF1 levels in Farage cells, but IL13 did not (Figure 5A). We next tested whether IL4 confers CHOP resistance to Farage cells. We treated Farage cells with CHOP or CHOP plus IL4 for 3 days. The combination of CHOP/IL4 significantly increased the IC_50_ compared with CHOP alone (Figure 5B, *p-value* < 0.0009). Addition of a lower IL4 concentration achieved a similar result (Supplementary Figure 2). IL4 suppressed apoptosis triggered by CHOP treatment (Figure 5C) and arrested the cell cycle at the G2/M phase (Figure 5D).

**Figure 6:**
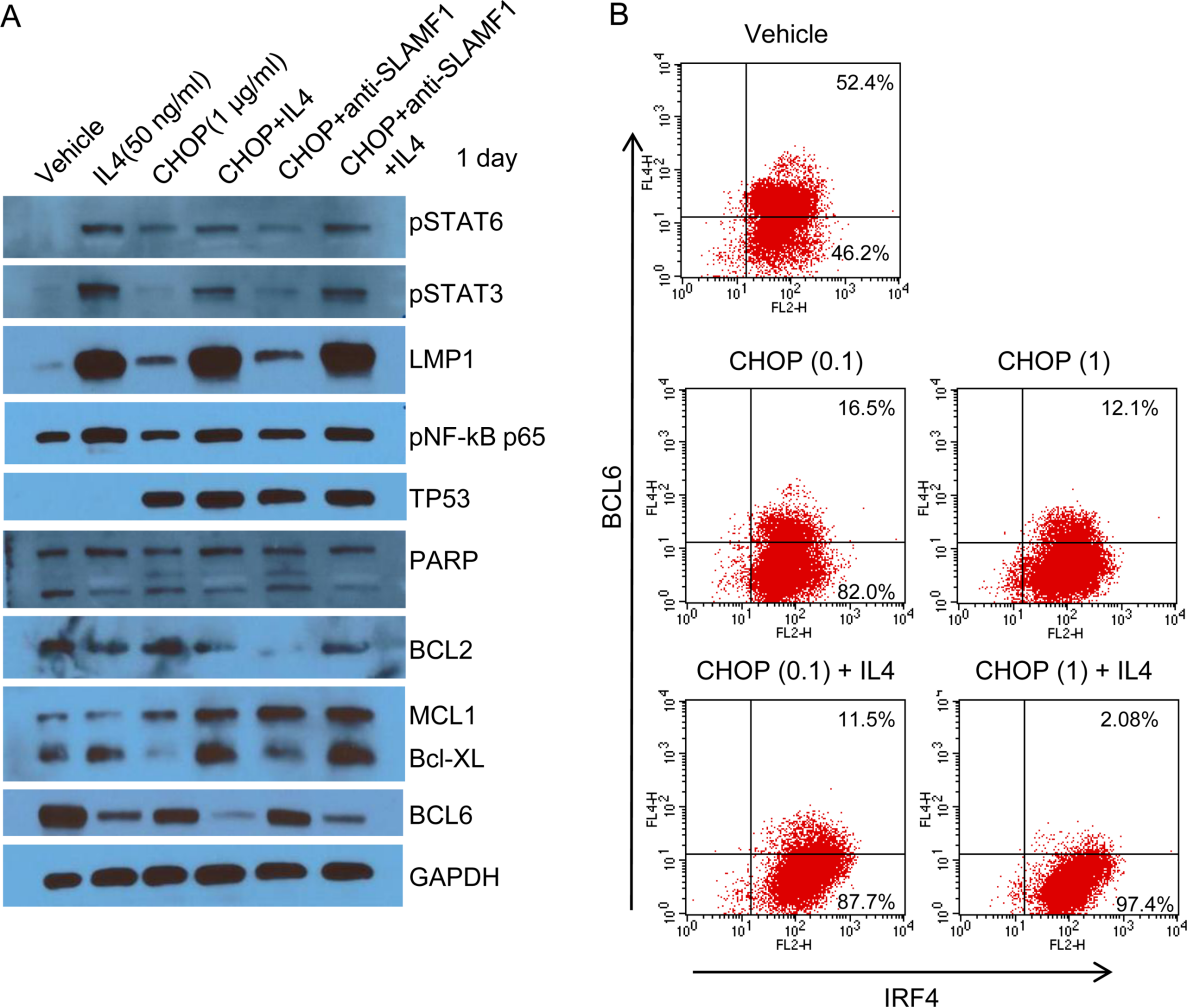
A large increase in LMP1 caused by IL4 suppressed CHOP-induced cell death (**A**) Farage cells were treated with 1 μg/mL CHOP alone or in combination with IL4 (50 ng/mL) or an anti-SLAMF1 antibody (500 ng/mL) for 2 days. The drug-treated cells were lysed, which was followed by SDS-PAGE and western blotting. The following antibodies were used: anti-SLAMF1, phospho-STAT3 (Tyr705), phospho-STAT6 (Tyr641), phospho-NF-κB p65 (Ser536), PARP, BCL-XL, MCL1, BCL6, Bcl-2, LMP1, TP53, and GAPDH. (**B**) Farage cells were treated with 0.1 or 1 μg/mL CHOP alone or in combination with IL4 (50 ng/mL). The drug-treated Farage cells were washed and incubated with an Fc blocker. To stain IRF4 and BCL6, the cells were fixed and permeabilized. Flow cytometry was then performed after incubating the cells with PE-conjugated anti-IRF4 or APC-conjugated anti-BCL6.

To investigate the underlying mechanism of the anti-apoptotic effect of IL4, we performed gene expression analysis and western blots. Of the genes associated with the IL4 pathway, AICDA, BCL2L1, CCL17, LTA and PDCD1LG2 were increased in the IL4+CHOP-treated cells as compared with the CHOP-treated cells (Supplementary Figure 3). The expression of BCL6, IL10, CD79A, SHC1, MYC and EGR1 were decreased (Supplementary Figure 3) in the IL4+CHOP-treated cells. We next performed western blot analysis for LMP1, phospho-STATs, pNF-κB (p65), TP53, c-Myc, BCL6 and anti-apoptotic proteins after 1 day of exposure to CHOP (1 μM) plus IL4 (50 ng/ml). Phosphorylated STAT3, STAT6, NF-κB and LMP1 were clearly increased in the IL4-treated cells (Figure 6A). In particular, LMP1 and pSTAT3 were greatly increased by IL4. Increase of TP53 was associated with CHOP treatment (Figure 6A), which indicated activation of the DNA damage signaling pathways. Of the three anti-apoptotic genes BCL2, MCL1 and BCL-XL, MCL1 and BCL-XL exhibited increased protein levels in agreement with the gene expression results (Figure 6A and Supplementary Figure 3). Expression levels of BCL2 appeared to be inversely correlated with those of the other anti-apoptotic genes. The BCL6 and c-Myc proteins were decreased (Figure 6A), which was indicated in the gene expression data. We confirmed the decrease in BCL6 at the single-cell level using FACS analysis (Figure 6B). The number of cells expressing BCL6 were decreased following CHOP treatment and further decreased following CHOP+IL4 treatment. LMP1 is known to be transactivated by host STAT3 or 6 as well as viral EBNA2 [21–22]. This finding suggests that EBNA2 might contribute to the great increase of LMP1 after IL4 treatment. Therefore, we examined whether CHOP or IL4 treatment exerted effects on EBNA2 expression using western blotting. EBNA2 was hardly expressed in the Farage cells and not further increased by either IL4 and CHOP or CHOP alone (Supplementary Figure 4), which lowers the potential dependency of LMP1 expression on EBNA2 in Farage cells. As a result, the significant increase in LMP1, pNF-κB (p65) and pSTAT3 following IL4 treatment might confer a CHOP-resistant molecular phenotype.

### Farage cells are sensitive to NF-κB and STAT inhibitors

**Figure 7:**
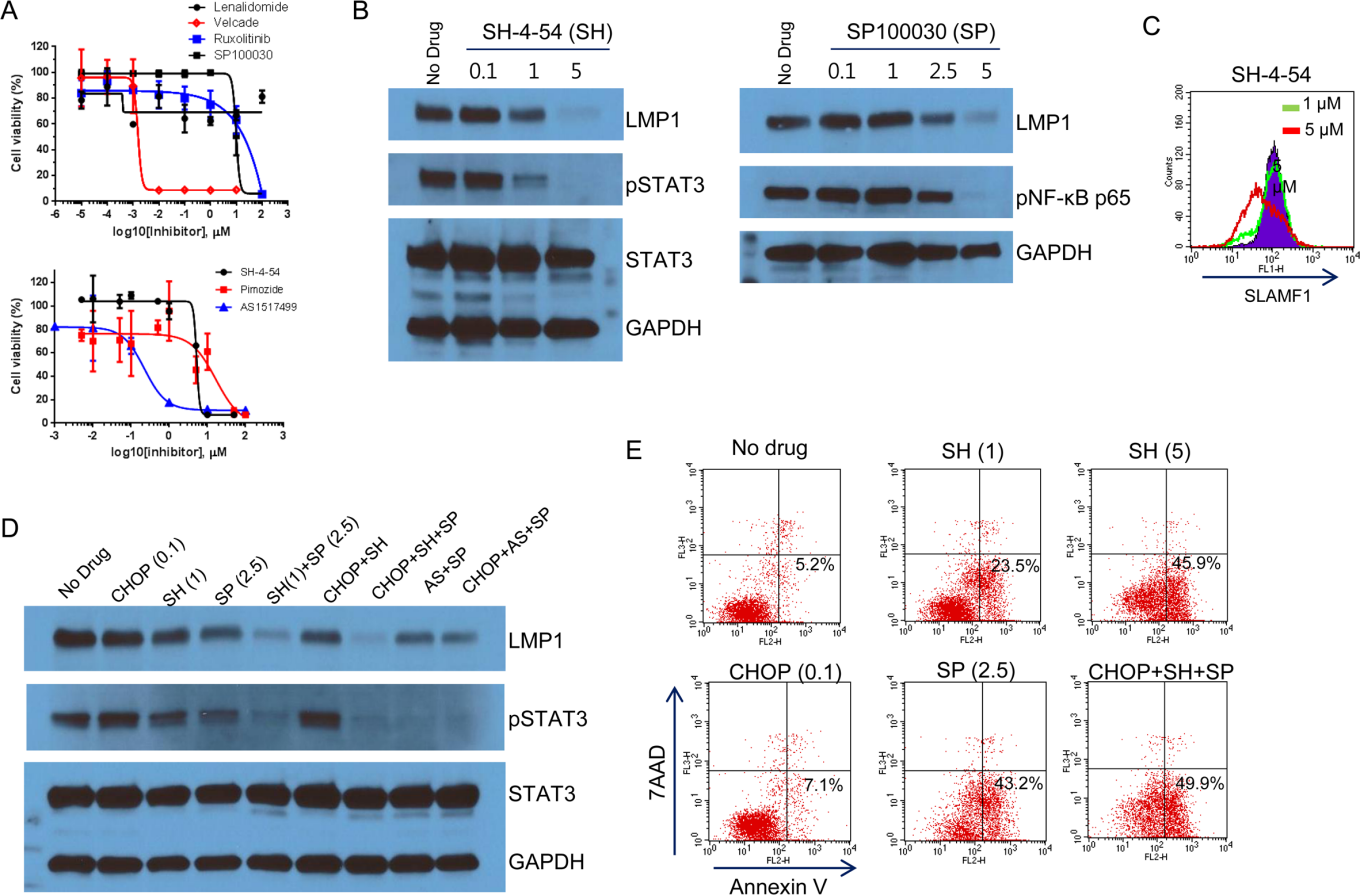
Inhibition of the NF-κB and STAT pathways (**A**) Farage cells were treated with various concentrations of lenalidomide, Velcade, ruxolitinib, SP 100030, SH-4-54, pimozide, and AS 1517499. Cell viability was measured using the WST-1 reagent and the determination of IC_50_ values and generation of the graphs were performed using GraphPad software. The IC_50_ values of Velcade and AS 1517499 were 0.0015 and 0.22 μM, respectively. (**B**) Farage cells were treated with 0.1, 1, or 5 μM SH-4-54 (left panel) or 0.1, 1, 2.5, or 5 μM SP 100030 (right panel) for 3 h. The cells were lysed followed by western blotting with anti-LMP1, pSTAT3, and pNF-κB p65 (Ser536) antibodies. (**C**) Farage cells were treated with 0, 1, or 5 μM SH-4-54 for 3 h. The cells were labeled with FITC-conjugated control IgG or anti-SLAMF1 antibodies and were subjected to FACS analysis. (**D**) Farage cells were treated with 0.1 μg/mL CHOP, 1 or 5 μM SH-4-54 (SH), 2.5 μM SP 100030 (SP), 1 μM AS 1517499 (AS), and combinations as indicated for 3 h. The cells were lysed followed by western blotting with anti-LMP1 and pSTAT3 antibodies. (**E**) Inhibition of pSTAT3 triggered early apoptosis in Farage cells. Farage cells were treated with 0.1 μg/mL CHOP, 1 or 5 μM SH-4-54 (SH), and 2.5 μM SP 100030 (SP) alone and in combination for 3 h. Annexin V-positive labeling (lower right) indicates cells undergoing early apoptosis.

The anthracycline-based CHOP is designed to trigger cell death by inducing DNA damage, but not by targeting the NF-κB or JAK/STAT signaling pathways. We therefore tested the sensitivity of Farage cells to drugs that target the NF-κB or JAK/STAT signaling pathways. We treated the Farage cells with the proteasome inhibitor Velcade (also known as bortezomib), which inhibits NF-κB activity [37], the AP1/NF-κB double inhibitor SP100030, the IRF4 TNF-α secretion inhibitor lenalidomide, the JAK1/2 inhibitor ruxolitinib, and STAT3, 5, and 6 inhibitors (SH-4-54, pimozide, and AS1517499, respectively). The Farage cells were most sensitive to Velcade, followed by the STAT6 and 5 inhibitors (Figure 7A). The IC_50_ values of Velcade and AS1517499 were 0.002 and 0.22 μM, respectively. The high sensitivity of the Farage cells to Velcade reflects their high dependency on constitutive NF-κB activation for cell survival, which was demonstrated in EBV+ B cells with type III latency [37]. Direct inhibition of STATs appeared to be more effective than JAK1/JAK2 inhibition in the Farage cells.

We next investigated whether these NF-κB and STAT inhibitors perturb the regulatory loop of LMP1 and whether this eventually leads to decreased LMP1 levels. We treated Farage cells with SH-4-54 and SP100030 alone or in combination (Figure 7B). Three hours after treatment, SH-4-54 and SP100030 clearly inhibited the phosphorylation of STAT3 and NF-κB at 1 or 2.5 μM, respectively (Figure 7B). LMP1 levels were decreased similarly to the levels of pSTAT3 or pNF-κB p65 (Figure 7B). SLAMF1 at the cell surface was also decreased when LMP1 was nearly depleted (Figure 7B and 7C). A combination of SH-4-54 with SP100030 further decreased pSTAT3 and LMP1 levels (Figure 7D). Similar results were observed in combination with AS1517499 and SP100030 (Figure 7D). Inhibition of pSTAT3 and pNF-κB p65 was correlated with the number of apoptotic Farage cells (Figure 7E). Collectively, our results suggest that the combination of NF-κB and STAT inhibitors is effective for inducing cell death in EBV+ DLBCL cells.

## DISCUSSION

LMP1 is a suggested driver of lymphomagenesis [9] and can activate multiple pathways, including the NF-κB signaling pathways that regulate cell survival [17–18]. We assumed that differential drug responses at the single-cell level may be responsible for the varying levels of LMP1. To indirectly isolate LMP1-positive live cells we used a FITC-conjugated anti-SLAMF1 antibody. We found that the expression level of LMP1 correlated well with that of SLAMF1 (Figure 1), and the SLAMF1^high^ Farage cells reflected the cellular and molecular characteristics of LMP1^high^ cells (Figures 1 and 2). The SLAMF1^high^ Farage cells were also enriched by CHOP or IL4 treatments. SLAMF1 is induced in activated normal B and T lymphocytes, dendritic cells and macrophages as well as EBV-infected lymphoblastoid cell lines (LCLs) [33, 38] and latency type III tumor cells [34]. However, the increase of SLAMF1^high^ cells by CHOP seemed to be unique to the tumor cells (Figure 3F). Expression of SLAMF1 is regulated by LMP1 through the NF-κB pathways [34]. However, the role of SLAMF1 as a downstream target of LMP1 remains unknown. We did not test diverse cell lines, but SLAMF1 appears to be a useful cell surface marker for the isolation of LMP1^high^ cells.

Patients with EBV+ or EBV- DLBCL have received a similar combination chemotherapy called CHOP or R-CHOP (rituximab combined with CHOP). However, EBV+ cases have a worse prognosis than EBV-negative cases [1–2, 5–8]. This result suggests that EBV infection-driven genetic or epigenetic alterations render tumor cells more resistant to the same chemotherapy. Gene expression profiles revealed that the activation of the NF-κB and/or JAK/STAT3 pathways are characteristics of EBV+ DLBCL, even when compared with ABC type EBV- DLBCL [4, 10]. Given that constitutive NF-κB and STAT3 activation obstructs with apoptotic effect of chemotherapy, activation of these pathways may be responsible for the poor outcomes observed after CHOP treatment in EBV+ DLBCL.

Indeed, cells that survived after CHOP treatment overexpressed genes regulated by NF-κB (Supplementary Table 2), suggesting activation of this pathway. However, this result was not fully supported by western blot. Phosphorylated NF-κB p65 or p100 were not significantly increased by a 24-hr exposure to CHOP, although LMP1 levels clearly increased (Figure 6A). About 2-fold increases in the phosphorylated NF-κB p65 and p100 were observed only after a substantial increase in LMP1 following the addition of IL4 (Figure 6A). This finding indicated that negative regulators of NF-κB may be activated to maintain a sustained level of activated NF-κB. Of the known negative regulators of NF-κB, USP18 and TNFAIP3 (A20) were overexpressed in CHOP-treated cells 3.8-fold and 1.5-fold, respectively. Recently, USP18 was identified as a negative regulator of NF-κB signaling by targeting TAK1 and NEMO for deubiquitination [39]. Overexpression of the chemokines CCL17 and CCL22 is an important signature of LMP1-mediated NF-κB activation, which has been demonstrated both *in vitro* and *in vivo* [28, 40]. CCL22 was always overexpressed in the SLAMF1^high^ Farage cells, whereas CCL17 was highly overexpressed in the presence of IL4 (Supplementary Figure 3), indicating that transactivation of CCL17 may require higher levels of NF-κB or co-activators associated with IL4. We suggest that the higher constitutive NF-κB activity in the LMP1^+^SLAMF1^high^ Farage cells confers higher resistance to CHOP.

LMP1 expression is induced by cytokines, such as IL10, IL-15, INF-γ, IL-13, and IL4 [21–22, 41], because its promoter contains STAT binding sites. Here, we observed that IL4 up-regulated STAT3, STAT6, and LMP1 in Farage cells (Figure 6A), which rendered the Farage cells resistant to CHOP. IL4 regulates lymphocyte differentiation, proliferation, and survival through the JAK/STAT6 or STAT3 and IRS-1/IRS-2/AKT pathways [36]. Lu et al. reported that DLBCL cells respond differently to IL4 according to the cell of origin [42]. Expression of IL4-inducible genes such as BCL6 was significantly higher in GCB-like DLBCL cells than in ABC-like DLBCL cells [42]. The opposite effects of IL4 on cell proliferation were observed according to the cell of origin. IL4 stimulated the proliferation of GCB-like DLBCL cells, but inhibited the proliferation of ABC-like DLBCL cells [42]. Similarly, EBV+ DLBCL may respond differently to IL4 than EBV- DLBCL because of the up-regulation of LMP1 by IL4, which we observed in the Farage cells. IL4 stimulated the proliferation of the Farage cells, but the Farage cells were ostensibly arrested in the G2/M phase by CHOP plus IL4 treatment (Figure 5). In Farage cells, BCL6 is highly expressed and was found to be quite stable against degradation because of the homozygous deletion of FBXO11 that controls the ubiquitination and degradation of BCL6 [43]. However, BCL6 levels were decreased after CHOP or IL4 treatment and further decreased by both CHOP and IL4 treatment in the Farage cells (Supplementary Figure 3 and Figure 6A and 6B). In contrast, IRF4 and PRDM1 or XBP1 were up-regulated by CHOP or/and IL4 in conjunction with NF-κB activation. Considering that NF-κB-mediated IRF4 induction downregulated BCL6 expression [44], the increased IRF4, possibly caused by IL4/STAT6 or STAT3/LMP1/NF-κB signaling, may lead to down-regulation of BCL6, resulting in the activation of the molecular program of post-germinal plasmablast cells. The surviving Farage cells after the CHOP+IL4 treatment appeared to be ABC-like DLBCL cells. Collectively, our data suggest that the presence of IL4 in the tumor microenvironment of EBV+ DLBCL may alter the outcome of CHOP treatment towards a poor prognosis.

In conclusion, higher activation of the NF-κB and JAK/STAT signaling pathways is a feature of EBV+ DLBCL and CHOP-resistant Farage cells. NF-κB and STAT inhibitors suppressed the viral oncogene LMP1 and cell survival. Therefore, a new combination therapy that includes NF-κB and STAT inhibitors may be more successful at eliminating EBV+ DLBCL cells.

## MATERIALS AND METHODS

### Cell lines and cell culture

Jijoye, IM-9, Daudi, and lymphoblastoid cell lines were obtained from the Korean Cell Line Bank (KCLB) (Seoul, South Korea) and Raji and Farage cell lines from the American Type Culture Collection (ATCC, Manassas, VA, USA). All cells were cultured in RPMI-1640 media supplemented with 10% heat-inactivated fetal bovine serum (ThermoFisher Scientific, Waltham, MA, USA) and antibiotics (100 μg/mL penicillin and streptomycin). All cells were grown in an incubator at 37°C under a 5% CO_2_ humidified atmosphere.

### Cell sorting

Farage cells were seeded at a density of 1 × 10^7^ cells in a T75 flask and cultured for 2 days. The cells were then washed, their Fc receptors were blocked, and SLAMF1 was labeled with FITC-conjugated anti-human SLAMF1/CD150 antibody (Cat# 306306, Biolegend, San Diego, CA, USA). The labeled cells were analyzed by a Becton-Dickinson FACS Aria III (Franklin Lakes, NJ, USA), and the top 10% most brightly labeled, moderately labeled, and bottom 10% dimly labeled cells were selected and designated as SLAMF1^high^, SLAMF1^inter^, and SLAMF1^low^, respectively. Subsequent analyses were performed as described below.

### Cell proliferation and viability test

Cells were seeded at a density of 2 × 10^4^ cells in 96-well plates for the cell proliferation and viability tests. In the drug treatment conditions, drugs were added 1 day following seeding, and the cells were cultured for 3 days. Cell proliferation and viability were measured using the WST-1 reagent according to the manufacturer's protocols (Roche, Indianapolis, IN). Optical density was measured at 450 and 600 nm 4 hr after adding the WST-1 reagent. The half maximal inhibitory concentration (IC_50_) was calculated with GraphPad software (La Jolla, CA, USA).

### Cell cycle analysis

FACS-sorted and drug-treated cells were washed with cold 1 × PBS and then fixed with 70% cold ethanol for more than 30 min at −20°C. Cells were washed with 1 × PBS and incubated with a staining solution including 50 μg/ml propidium iodide (PI) and 200 μg/ml RNase A for 15 min at 37°C. DNA content was analyzed using a Becton-Dickinson FACS Calibur flow cytometer. Data were analyzed with CellQuest software (Becton Dickinson, Heidelberg, Germany).

### Drug inhibition experiments

CHOP consisted of four drugs (cyclophosphamide, vincristine, adriamycin, and prednisone) in a ratio of 80/5.5/0.16/11.1, respectively [45]. All four drugs were purchased from Selleckchem (Houston, TX, USA). Farage cells were treated with various concentrations of CHOP for 2 days or 3 days. Other drugs and their sources were as follows: Velcade (Selleckchem), ruxolitinib (Axon Medchem, Groningen, Netherlands), lenalidomide (Axon Medchem), SP 100030 (Tocris Bioscience, Bristol, United Kingdom), pimozide (Calbiochem, Darmstadt, Germany), AS 1517499 (Axon Medchem) and SH-4-54 (Selleckchem). The determination of IC_50_ and generation of the graphs were accomplished using GraphPad software.

### Enzyme-linked immunosorbent assay (ELISA) for IL10 and CCL22

To detect secreted CCL22, Farage cells were sorted into three groups (SLAMF1^high^, SLAMF1^inter^, SLAMF1^low^) as described above. We then seeded the sorted cells into 96-well plates and cultured them for 24 hr and used the culture media for ELISA. We performed ELISA (ELISA MAX™ Deluxe Set) according to the manufacturer's protocol (R&D Systems, Minneapolis, MN, USA). To detect secreted IL10 after CHOP treatment, Farage cells were seeded at 1 × 10^6^ cells in a T25 flask. The next day, the cells were treated with various concentrations of CHOP for 24 hr. The CHOP-treated cells were seeded at 5 × 10^4^−1 × 10^5^ cells and incubated for 24 hr without CHOP. The culture medium was removed, and ELISA was performed by using an ELISA kit (R&D Systems). Cell numbers were normalized by the cell viability test.

### Analysis of apoptosis

Drug-treated Farage cells were stained with Annexin V-fluorescein isothiocyanate (FITC) (BD Biosciences, Heidelberg, Germany) and propidium iodide (PI) (Sigma Aldrich, St. Louis, MO, USA) and analyzed by flow cytometry. Detection of early apoptotic (Annexin V-FITC+ and PI-) and late apoptotic cells (Annexin V-FITC+ and PI+) was performed using a FACS Calibur (Becton and Dickinson). Data were analyzed with CellQuest software (Becton Dickinson).

### Western blot analysis

Farage cells were treated with 0.01, 0.1, 0.5, and 1 μg/ml CHOP alone or in combination with 1 μg/ml CHOP and IL4 (cat# 574002, Biolegend, 50 ng/ml) or 0.1 μg/ml CHOP and various concentration of AS 1517499. The FACS-sorted and the drug-treated cells were lysed with M-PER buffer (Pierce Biotechnology, Rockford, IL) containing a 1× protease and phosphatase inhibitor cocktail (Roche). Then, 20–40 μg of lysate was separated on 4–15% or 12% precast sodium dodecyl sulfate-polyacrylamide gels and transferred onto polyvinylidene fluoride membranes (Bio-Rad Laboratories, Hercules, CA). The membranes were blocked with 5% non-fat dry milk and incubated with appropriate primary and secondary antibodies. Signals were detected using the SuperSignal West Pico Chemiluminescent Substrate (Pierce Biotechnology). The following antibodies were used in this study: phospho-NF-kB p65 (3033), PARP (9542), BCL-XL (2764), MCL1 (5453), BCL6 (14895), caspase-3 (9668), Bcl-2 (4223), phospho-STAT3 (9145), phospho-STAT5 (4322), and phospho-STAT6 (9361) were purchased from Cell Signaling Technologies (Beverly, MA); and LMP1 (CS1-4, M0897) from Dako (Glostrup, Denmark); TP53 (sc-126), GAPDH (sc-25778), and goat anti-rabbit IgG (sc-3837) from Santa Cruz Biotechnology (Santa Cruz, CA, USA); we developed an anti-SLAMF1 rabbit polyclonal antibody for western blotting.

### Flow cytometry for the detection of transcription factors

For staining cell surface markers, 1 × 10^6^ cells were washed in 1 × PBS buffer and resuspended in 100 μl FACS buffer. To block the Fc receptor, 5 μl of TruStain (Biolegend) was added and incubated on ice for 15 min. The cells were then washed and resuspended in 100 μl FACS buffer. FITC-conjugated anti-CD10 or SLAMF1 was added (2.5–5 μl) and incubated at room temperature for 30 min. For staining IRF4 and BCL6, we followed the methods described by Albu et al. [46]. Briefly, the cells were washed and incubated with an Fc blocker. The cells were then fixed with fixation buffer (3% formaldehyde, 0.1% saponin in PBS) and permeabilized with permeabilization buffer (1% FBS, 0.5% saponin, 10 μg/ml RNase A in PBS) before incubation with PE-conjugated anti-IRF4 (646403) or APC-conjugated anti-BCL6 (358505). Isotype control APC mouse IgG2b (400319) and PE mouse IgG2a (400213) served as controls. All antibodies were purchased from Biolegend. Flow cytometry was performed on a FACS Calibur, and data were analyzed using CellQuest software (BD Biosciences).

### Gene expression profiling and data analysis

We generated gene expression profiles from three different experimental conditions using the Affymetrix GeneChip HuGene 2.0 ST oligonucleotide arrays (Affymetrix, Santa Clara, California). The three experimental conditions were: 1) comparison of SLAMF1^high^ and SLAMF1^low^ cells; 2) Farage cells treated with 1 μg/ml CHOP for 2 days and 3 days compared with untreated Farage cells; 3) Farage cells incubated with 1 μg/ml CHOP plus 50 ng/ml IL4 compared with 50 ng/ml IL4 alone for 2 days. Total RNA was extracted from the harvested cells using an RNeasy kit (QIAGEN, Germany) and were sent to the local Affymetrix GeneChip service provider (DNA LINK, Seoul, Korea). One hundred nano-grams of high quality total RNA was converted to double-stranded cDNA. Subsequent amplification of cRNA, labeling of probes, and scanning signals were performed according to the company's protocol. Gene expression estimates were normalized using the robust multiarray averaging (RMA) method. The microarray data followed the MIAME guidelines and were deposited in the GEO database (GSE81413). Genes showing an average fold-change greater than 2.0 (*p <* 0.05, unpaired *t-test*) between the SLAMF1^high^ and SLAMF1^low^ cells or between given experimental conditions were considered to be significantly differentially expressed. The differentially expressed genes were subjected to functional annotation clustering analysis using the Database for Annotation, Visualization, and Integrated Discovery (DAVID) (http://david.abcc.ncifcrf.gov/home.jsp) or gene set enrichment analysis (GSEA) (http://software.broadinstitute.org/gsea/index.jsp).

### Statistical analysis

All data were analyzed with the statistical program, GraphPad Prism 6.0 (San Diego, CA). Student's *t-test* was used to determine the statistical significance of differences between groups.

## SUPPLEMENTARY MATERIALS FIGURES AND TABLES


