# Supplementary material for: LMP1+SLAMF1high cells are associated with drug resistance in Epstein-Barr virus-positive Farage cells
Source: Oncotarget. 2017 Feb 21;8(15):24621–34. doi: 10.18632/oncotarget.15600 (PMC5421874; doi:10.18632/oncotarget.15600)
Supplement: Supplementary file 1 [file oncotarget-08-24621-s001.pdf]

LMP1^{+}SLAMF1^{high} cells are associated with drug resistance in Epstein-Barr virus-positive Farage cells

Supplementary Materials

Supplementary Table 1: Gene set analysis of overexpressed genes in SLAMF1^{high} Farage cells

GO Term	Genes	*p*-value	FDR (%)
M phase of mitotic cell cycle	KIF23, CDK1, DLGAP5, CCNF, KIF18A, NDC80, CENPE, ANLN, ESPL1, UBE2C, FAM83D, CDCA8, PLK1, INCENP, BUB1, SKA3, ASPM	3.02E-16	5.00E-13
mitotic cell cycle	KIF23, CDK1, DLGAP5, CCNF, KIF18A, ESPL1, NDC80, CENPE, ANLN, UBE2C, GTSE1, FAM83D, CDCA8, CCND2, PLK1, INCENP, BUB1, SKA3, ASPM	2.75E-15	4.15E-12
chromosome segregation	HJURP, DLGAP5, INCENP, KIF18A, SKA3, CENPE, NDC80, ESPL1, TOP2A	1.13E-09	1.68E-06
regulation of mitotic cell cycle	CDK1, DLGAP5, BUB1, ANLN, CENPE, ESPL1, UBE2C, GTSE1	2.54E-06	0.003
microtubule-based movement	KIF23, KIF18A, KIF18B, CENPE, KIF20A	0.001	1.68
apoptosis	CKAP2, IL2RA, LITAF, ESPL1, PDCD4, TOP2A, ECT2, TP53INP1	0.01	14.72
inflammatory response	CCL22, TNFSF4, IL2RA, CD44, TLR7	0.04	47.99

Supplementary Table 2: Gene set analysis of overexpressed genes in CHOP-treated Farage cells

Gene Set Name	Genes	*p*-value	FDR *q*-value
INTERFERON_ALPHA_ RESPONSE	STAT2 , TAP1, CXCL10, GBP4, CASP1, TXNIP, IFI44, L MX1, IFI44, USP18, SAMD9L, IFI35, SP110, HERC6, GBP2, SELL, LAMP3, MOV10, SAMD9, UBA7	6.64E-23	2.95E-21
INTERFERON_GAMMA_ RESPONSE	STAT2 , TAP1, CXCL10, GBP4, CASP1, TXNIP, IFI44L, MX1, IFI44, USP18, SAMD9L, IFI35, SP110, HERC6, IL2RB, CXCL9, STAT1, STAT4, TNFSF10, CD69, TNFAIP2, VAMP5, OAS2, OAS3, RNF213	1.18E-22	2.95E-21
ALLOGRAFT_ REJECTION	TAP1, IL2RB, CXCL9, STAT1, STAT4, GBP2, IL10, CCND2, IL2RA, TIMP1, NLRP3, CCL22, CXCL13, PRF1, IFNG, CFP, CD96, HLA-DOA, ITK, IL27RA	1.90E-16	2.38E-15
IL2_STAT5_SIGNALING	IL2RB, IL10, CCND2, IL2RA, CXCL10, GBP4, TNFSF10, SELL, TNFRSF1B, NFIL3, FURIN, MUC1, HIPK2, GLIPR2, IRF6, TLR7, SNX9, AHNAK, CAPN3, EOMES	1.90E-16	2.38E-15
INFLAMMATORY_ RESPONSE	IL10 , CXCL10, TNFSF10, SELL, TNFRSF1B, CXCL9, TIMP1, NLRP3, CCL22, CD69, LAMP3, IL7R, EDN1, ITGB3, EBI3, KIF1B, SEMA4D	3.93E-14	3.28E-13
TNFA_SIGNALING_VIA_ NFKB	CXCL10, CD69, IL7R, EDN1, NFIL3, TAP1, TNFAIP2, JUN, PLK2, DRAM1, CFLAR, AREG, PLAU, DUSP5, DUSP1, KLF2, SIK1, B4GALT5	3.93E-14	3.28E-13
P53_PATHWAY	TAP1 , JUN, PLK2 , DRAM1, CCND2, CASP1, TXNIP, FDXR, FUCA1, PLXNB2, S100A10, VDR, PHLDA3, SESN1, MDM2, ZMAT3, TCN2	5.16E-13	3.69E-12
IL6_JAK_STAT3_ SIGNALING	JUN, CXCL10, TNFRSF1B, CXCL9, ITGB3, EBI3, IL2RA, STAT1, CXCL13, STAT2, IL13RA1, IL10RB	4.51E-12	2.82E-11
APOPTOSIS	TAP1, CCND2, CASP1, TXNIP, FDXR, CD69, CFLAR, TNFSF10, TIMP1, PRF1	6.65E-08	3.70E-07
CHOLESTEROL_ HOMEOSTASIS	NFIL3, STARD4, CPEB2, ETHE1, GLDC, GSTM2, TP53INP1, JUN	1.92E-06	9.61E-06
EPITHELIAL_ MESENCHYMAL_ TRANSITION	TIMP1, ITGB3, FUCA1, AREG, FBN1, LGALS1, DST, FSTL3, PTHLH	4.52E-06	1.88E-05
KRAS_SIGNALING_UP	FUCA1, CCND2, CXCL10, TNFRSF1B, IL7R, PLAU, CXCR4, FCER1G, LY96, PRDM1	4.52E-06	1.88E-05

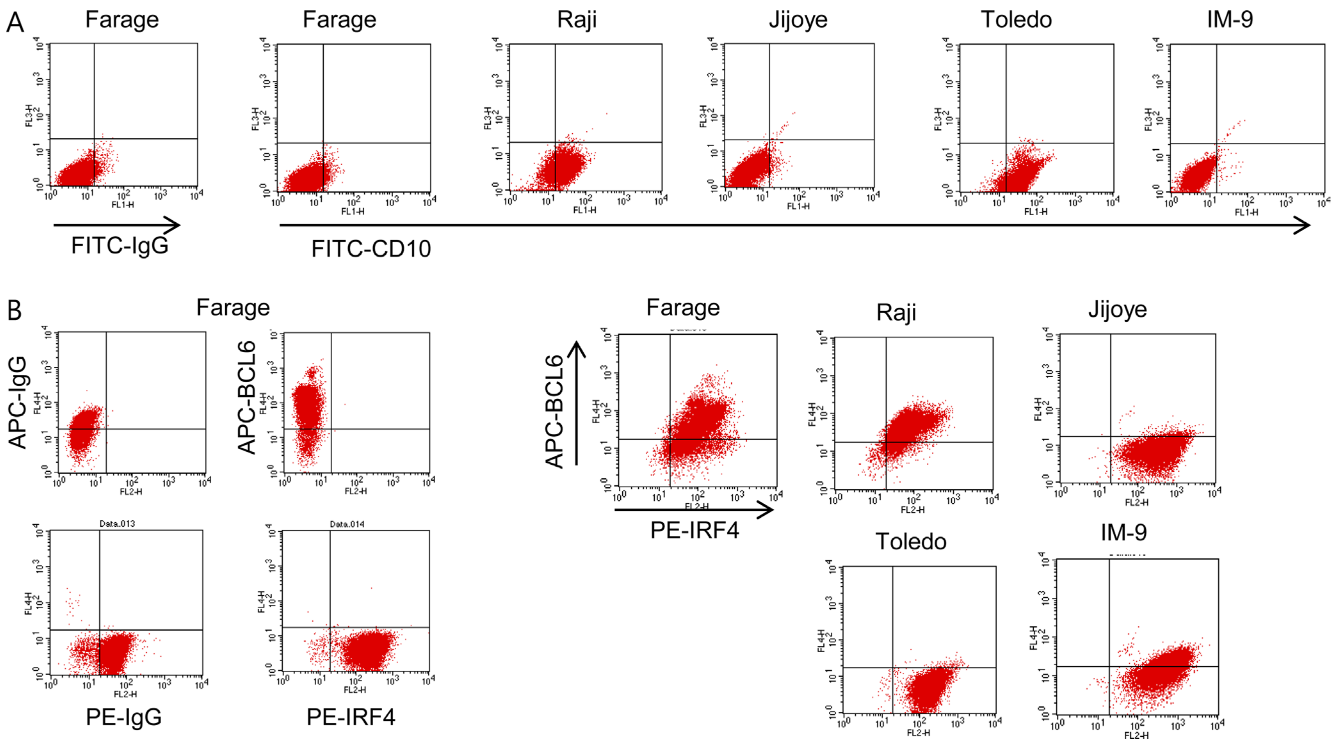

Supplementary Figure 1: Immunophenotype of EBV+ Farage cells. Expression levels of BCL6, IRF4/MUM1, and CD10 were analyzed using FACS (see Materials and Methods). Cells were washed followed by blocking of the Fc receptors with TruStain. The cells were then stained with FITC-conjugated anti-CD10 and analyzed by FACS (**A**). For the staining of IRF4 and BCL6 (**B**), the cells were washed and incubated with an Fc blocker. Next, the cells were stained with PE-conjugated anti-IRF4 or APC-conjugated anti-BCL6 following fixation and permeabilization. Flow cytometry was performed on a FACS Calibur, and the data were analyzed using CellQuest software (BD Biosciences).

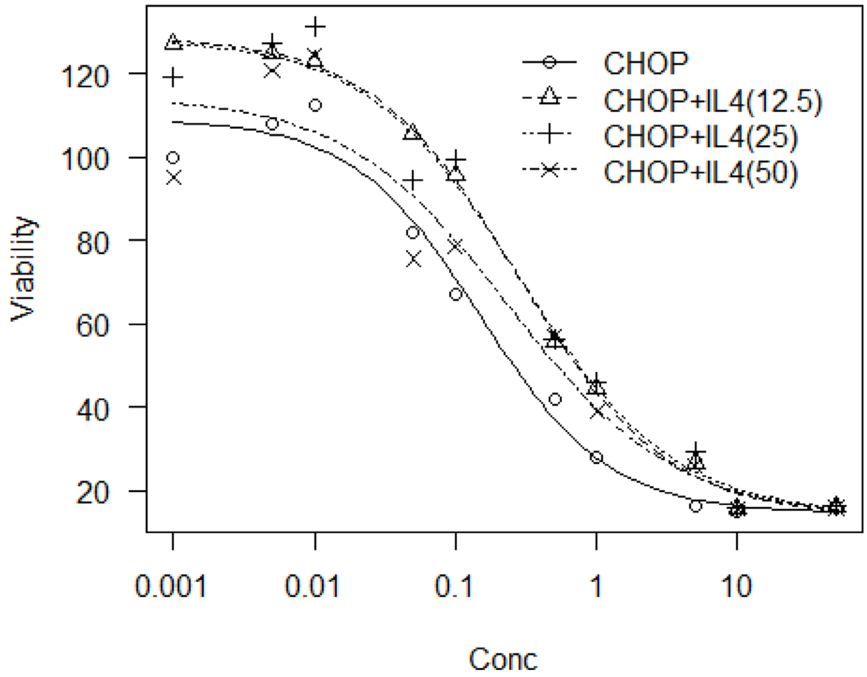

Supplementary Figure 2: Addition of IL4 increased cell viability against CHOP treatment. IL4 was added to culture media at concentrations of 0, 12.5, 25, and 50 ng/mL. Cell viability was measured 3 days after CHOP treatment. All concentrations of exogenous IL4 increased the IC50 values compared with CHOP alone (IC50 of CHOP alone and CHOP+IL4 (12.5 ng/mL): 0.15 and 0.30 µg/mL, respectively).

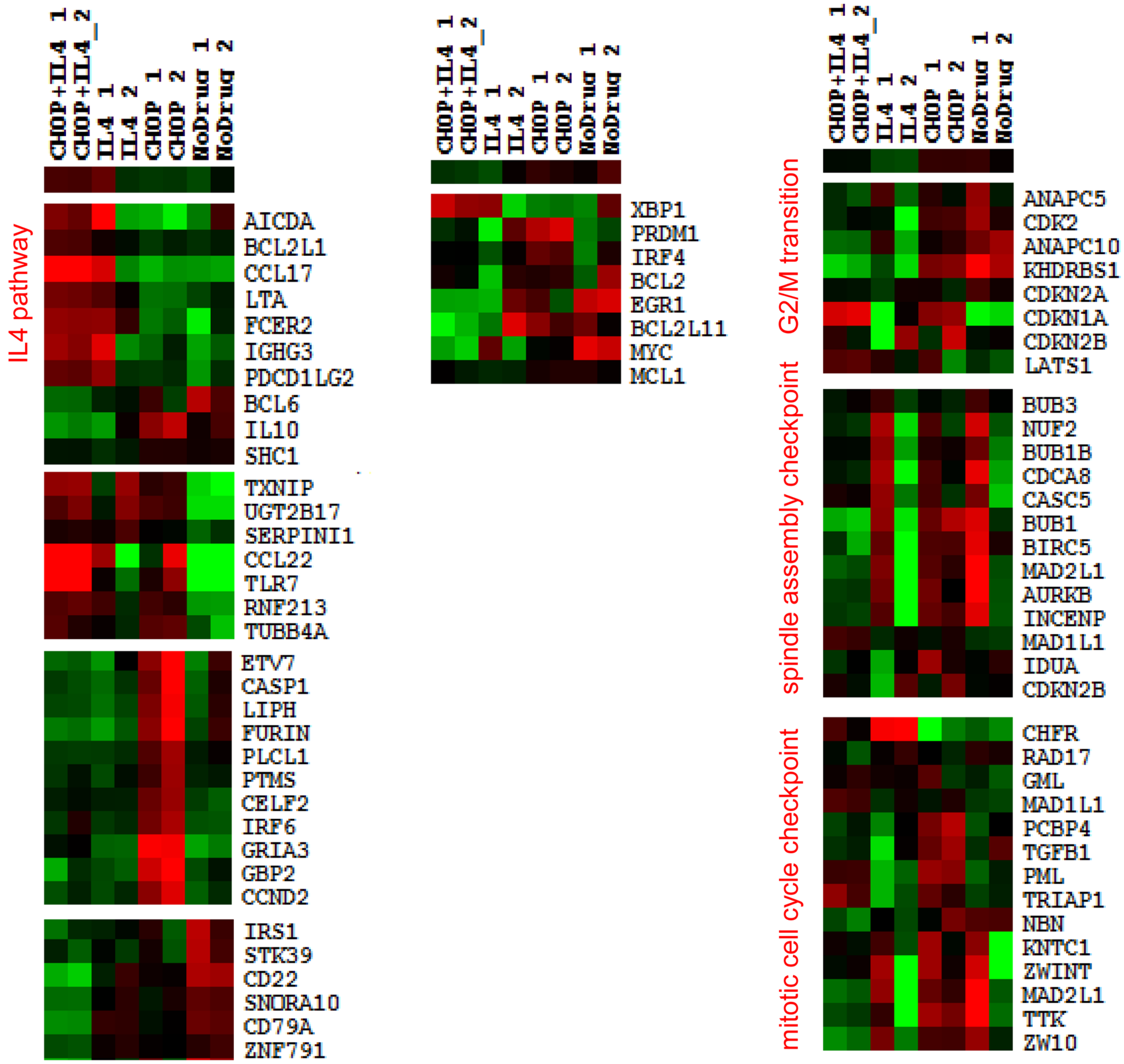

Supplementary Figure 3: Effect of IL4 on gene expression in Farage cells. Farage cells were incubated with 50 ng/mL IL4 alone or in combination with 1 µg/mL CHOP for 2 days. Gene expression profiles were generated with the Affymetrix GeneChip. Differentially expressed genes induced by IL4 were analyzed together with microarray data for CHOP only and NoDrug (the same data utilized for Figure 4 and Supplementary Table 2). AICDA, BCL2L1, and CCL17 were up-regulated, whereas CD79A, BCL6, and IL10 were downregulated by IL4.

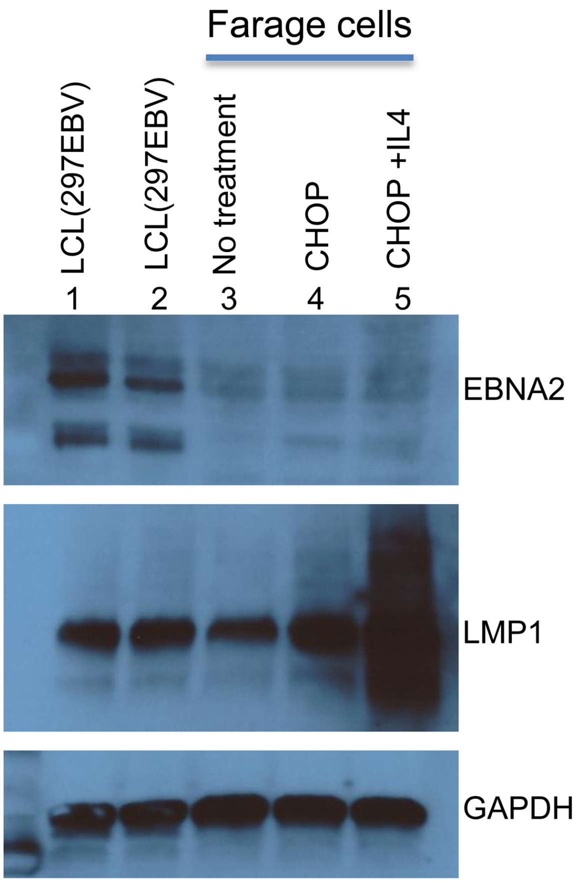

Supplementary Figure 4: EBNA2 was not increased by CHOP or CHOP+IL4 in Farage cells. The LCL (297EBV) cell line is an EBV-infected lymphoblastoid cell line that was purchased from the Korean Cell Line Bank (Seoul, South Korea). These LCL (297EBV) cells served as a positive control for the EBNA2 immunoblot. Forty and Twenty micrograms of cell lysate were loaded in the lanes 1 and 2, respectively. The same cell lysates used in the Figure 6A were applied for CHOP or CHOP+IL4 treated Farage cells (lanes 4 and 5). Western blots were performed for EBNA2, LMP1 and GAPDH. The anti-EBNA2 antibody was purchased from Abcam (ab90453).
